# Effects of Long-Term Physical Activity and Diet on Skin Glycation and Achilles Tendon Structure

**DOI:** 10.3390/nu11061409

**Published:** 2019-06-22

**Authors:** Joachim Nymann Hjerrild, Alexander Wobbe, Martin B. Stausholm, Anne Ellegaard Larsen, Christian Ohrhammer Josefsen, Nikolaj M. Malmgaard-Clausen, Flemming Dela, Michael Kjaer, S. Peter Magnusson, Mette Hansen, Rene B. Svensson, Christian Couppé

**Affiliations:** 1 Section for Sports, Department of Public Health, Aarhus University, 8000 Aarhus, Denmark; jnhjerrild@gmail.com (J.N.H.); alexander@wobtop.com (A.W.); anne2larsen@gmail.com (A.E.L.); mhan@ph.au.dk (M.H.); 2 Department of Physical and Occupational Therapy, Bispebjerg Hospital, 2400 Copenhagen, Denmark; mbsfys@gmail.com (M.B.S.); christianjosefsen@gmail.com (C.O.J.); p.magnusson@sund.ku.dk (S.P.M.); 3 Department of Global Public Health and Primary Care, University of Bergen, 5018 Bergen, Norway; 4 Institute of Sports Medicine Copenhagen, Department of Orthopaedic Surgery M, Bispebjerg Hospital and Center for Healthy Aging, Faculty of Health and Medical Sciences, University of Copenhagen, 2400 Copenhagen, Denmark; svensson.nano@gmail.com (R.B.S.); nikolajmoelkjaer@gmail.com (N.M.M.-C.); michaelkjaer@sund.ku.dk (M.K.); 5 Xlab and Center for Healthy Aging, Faculty of Health and Medical Sciences, University of Copenhagen, 2200 Copenhagen, Denmark; fdela@sund.ku.dk; 6 Department of Geriatrics, Bispebjerg-Frederiksberg University Hospital, Copenhagen University of Copenhagen, 2400 Copenhagen, Denmark

**Keywords:** skin autofluorescence, advanced glycation end-products (AGEs), ultrasound doppler, western diet, habitual exercise

## Abstract

Advanced glycation end-products (AGEs) accumulate with aging and have been associated with tissue modifications and metabolic disease. Regular exercise has several health benefits, and the purpose of this study was to investigate the effect of regular long-term exercise and diet on skin autofluorescence (SAF) as a measure of glycation and on Achilles tendon structure. In connection with the 2017 European Masters Athletics Championships Stadia, high-level male athletes (*n* = 194) that had regularly trained for more than 10 years were recruited, in addition to untrained controls (*n* = 34). SAF was non-invasively determined using an AGE Reader. Achilles tendon thickness and vascular Doppler activity were measured by ultrasonography, and diet was assessed by a questionnaire. There was no significant difference in SAF between the athletes and controls. However, greater duration of exercise was independently associated with lower SAF. Diet also had an effect, with a more “Western” diet in youth being associated with increased SAF. Furthermore, our data demonstrated that greater Achilles tendon thickness was associated with aging and training. Together, our data indicate that long-term exercise may yield a modest reduction in glycation and substantially increase Achilles tendon size, which may protect against injury.

## 1. Introduction

As part of the normal aging process, collagen-rich connective tissues, such as skin, cornea, cartilage, arteries, and tendon, undergo progressive and irreversible changes that are associated with tissue injury and several lifestyle diseases [1]. A major component of age-related impairment of these connective tissues are long-lived protein modifications derived from the formation of advanced glycation end-products (AGEs). AGEs are formed through a non-enzymatic reaction between reducing sugars and free amino groups of proteins, lipids, or nucleic acids that accumulate with age in long-lived molecules, including the collagen of skin and tendon [2,3]. Tendons consist almost entirely of type I collagen fibrils and are especially important in relation to sports because they are responsible for transmitting force from muscles to bones. The Achilles tendon is one of the largest and most heavily loaded tendons in the body and displays barely any turnover [4,5,6]. The Achilles tendon is also frequently injured during physical activity, either in the form of chronic pain (tendinopathy) or more rarely as an acute rupture [7,8]. Modification by AGEs can directly affect the collagen fibrils that make up connective tissues by cross-linking and swelling [9,10], and it has been demonstrated to reduce collagen fibril sliding [11,12] and cause impaired mechanics and fragility of connective tissues [13,14,15]. These tissue glycation changes may also be involved in macro-structural thickening on ultrasound and increased stiffness with aging and diabetes that may predict microvascular complications independent of Hb1AC [16,17,18,19].

Some AGEs are fluorescent and can be measured noninvasively by skin autofluorescence (SAF) [2]. SAF increases with age and is also greater in patients with certain chronic diseases, such as type 1 and 2 diabetes, where it reflects the glucose level over several years [2,20,21]. AGE accumulation in skin appears strongly linked to macro- and microvascular complications in diabetes and mortality, indicating that SAF reflects AGE modifications of other long-lived tissues [20,21,22]. Aside from accumulating in vivo, AGEs also form between sugars and proteins during food processing, and in recent years, there has been much focus on the negative effects of dietary intake of AGEs on healthy tissues. There is some debate about the primary pathway by which diet may influence AGEs in vivo [23], whether AGE-modified dietary amino acids are absorbed and find their way into tissues or dietary reducing sugars react with endogenous proteins to form new AGEs. Current evidence supports that ingestion of AGE-rich diet increases the AGE content in many of the aforementioned connective tissues and organs leading to age-related changes, and pathology in both animals and humans [13,14,24,25,26,27]. Of note, the consumption of coffee and meat has been associated with higher SAF [28,29]. Long distance runners have lower glycation in tendon (pentosidine) and in skin (SAF) compared to age-matched sedentary controls [16], which could explain why aging runners have reduced disability and mortality compared to sedentary persons [30]. Moreover, life-long endurance athletes have larger tendons than both their age-matched sedentary controls and young athletes, which may help prevent injury by reducing stress on the tendon [16]. However, the associations between exercise and aging on glycation (SAF), tendon size and vascularity have, to our knowledge, never been investigated in a larger cohort of healthy aging athletes.

This study, therefore, had two aims: (1) To investigate the effects of life-long physical activity on SAF and Achilles tendon structure, (2) to determine if SAF and tendon structure is influenced by dietary factors. The hypotheses were: (A) Long-term physical activity results in lower SAF. (B) Consumption of coffee and a “Western” diet pattern are associated with increased SAF. (C) Elevated SAF is independently associated with greater Achilles tendon thickness and Doppler activity. (D) Long-term physical activity results in greater Achilles tendon thickness and greater Doppler activity.

## 2. Materials and Methods 

### 2.1. Design

This is a cross-sectional study primarily including male athletes participating in the 2017 European Masters Athletics Championships Stadia (EMACS) in Aarhus, Denmark. The participants filled out a questionnaire including, amongst others, items on physical activity and diet. They had a SAF measurement to estimate skin glycation, their Achilles tendons were examined by ultrasound and biometric parameters were measured.

### 2.2. Participants

The study recruited 175 male athletes of 24 different nationalities at the 2017 EMACS. Additionally, 19 Danish male runners aged 22–41 were recruited through another running study at Aarhus University. Inclusion criteria were males at the age of 18 or older that had been training at least twice a week for the past 10 years and had not been doing strenuous exercise 24 h prior to investigation. Exclusion criteria were current smoking and diabetes, based on self-report. Participants were recruited through bulletins, flyers and actively contacted at the event. At initial contact, participants were screened against the eligibility criteria. In addition to the athletes, a group of Danish sedentary controls (*n* = 34) were subsequently recruited through social media. The eligibility criteria were the same as for the athletes, except the controls were required to not have performed regular exercise for the past 10 years. Participants were purposely recruited in a wide age range in both the athletic and control group. A number of participants were excluded after inspecting the questionnaire replies (see below). Eight EMACS participants and four of the Danish runners were excluded (five due to smoking, seven due to less than 10 years of training), leaving 167 EMACS participants and 15 Danish runners in the athlete group.

The study was approved by the Danish ethical committee of The Capital Region, registry number: H-3-2014-017-58734. All participants gave their informed consent to participate before the investigation.

### 2.3. Questionnaire

Participants completed a questionnaire (see Supplementary Materials) containing information on the current amount of exercise (weekly hours at <70% max heart rate, weekly hours at >70% max heart rate, training years), diet (fruit, vegetables, fish, bread, cereals, coffee, wine, beer, liqueurs, total fluid, pure water) as well as overall diet pattern (“Western” versus “Mediterranean”) currently and during youth, and finally smoking. The diet part of the questionnaire assessed food frequency over the past three months in fixed ranges of either weekly or daily intake (see Supplementary Materials). For analyses, these ranges were parameterized to weekly intakes based on the average of the range. The item on overall diet pattern involved estimating the relative percentage of the diet that was predominantly “Western” vs. “Mediterranean”, based on a description of food items characterizing the diet (see Supplementary Materials). The sum of the two was required to be 100%. The “Western” diet is characterized by a high content of meat, fat, sugar and processed foods including soft drinks, whereas the “Mediterranean” diet is lower in meat and rich in plants, unsaturated fats, and unprocessed foods. This assessment was made both for current diet over the past three months, as well as during the first 0–17 years of life.

In addition to these questions, the questionnaire also contained items on specific sports disciplines with more detailed quantification of activity levels, past sports-related injuries to the lower body, present symptoms/pain in the Achilles tendon, disease, medication, dietary supplements, and sleep. However, to reduce complexity these items were not included in the analyses of the present study.

### 2.4. Skin Autofluorescence

Some AGEs are naturally fluorescent such as pentosidine [31] and in this study, we estimated the amount of AGEs in the skin using an AGE Reader (DiagnOptics B.V., Groningen, The Netherlands), which non-invasively measures skin autofluorescence (SAF). This method has been comprehensively described and validated, including by comparison of SAF results with skin biopsy-analysis in different populations (diabetes, renal failure, and controls) [2].

The AGE Reader illuminates approximately 1 cm^2^ of the skin with excitation wavelengths of 300–420 nm. SAF is calculated using the ratio between light intensity in the wavelength range of 420–600 nm and the average reflected intensity in the wavelength range of 300–420 nm. The result is given in arbitrary units on the range of zero to five where higher values represent greater amounts of AGEs [2].

Measurements were performed on the volar side of the participants’ non-dominant forearm. None of the participants reported to have used skin care products prior to the examination, but as a precaution, the area of measurement was cleaned using alcohol swabs to prevent any skin lotion etc. from affecting the AGE readers result. Each participant was measured at three neighboring locations on the skin, in a seated position at room temperature and the median value was used for further analyses.

### 2.5. Ultrasonography

A portable ultrasound machine (uSmart 3200T, Terason, Burlington, MA, USA) with a 16-5 MHz probe (16L5 Smart Mark, Terason, Burlington, MA, USA) was used to measure the anteroposterior thickness of the Achilles tendon at both the proximal and distal end of the free tendon, and to measure blood flow within the tendon by Doppler. Prior to the study, an optimized set of imaging parameters were determined to maximize power Doppler sensitivity with minimum noise. All images were recorded using these settings. Anatomical images were obtained at 30 mm depth of view, frame rate 11 Hz, B-mode gain 53, dynamic range 70 and with tissue harmonic imaging (THI) on. Power Doppler was overlaid on the anatomical images in a box covering the full image width and 15 mm depth with the following parameters: Ultrasound frequency 6.6 MHz, Doppler gain 47, pulse repetition frequency 0.6 kHz and a wall filter of 21 Hz. It was important that the participants did not perform any strenuous exercise within 24 h prior to examination since there is a trend toward greater Doppler activity among athletes who have been active before imaging [32].

Imaging was performed in the sagittal plane with the participant in a prone position with both feet hanging over the end of the examination table. The distal area was imaged first at the Achilles attachment to calcaneus, and then the probe was displaced by one probe-width to scan the proximal area containing the soleus insertion, without overlapping the distal scan. Thickness was measured at the thickest point of the tendon and vascularity was quantified as the area covered by Doppler signal within the tendon. The average value for the proximal and distal region was determined for each leg, and the two legs were averaged for use in the analyses. Four participants had a previous Achilles tendon rupture on one leg, and for these only the healthy leg was included in ultrasound analyses.

### 2.6. Statistics

Differences between athletes and controls in basic characteristics and diet were analyzed by Welch’s t-tests. Relationships between SAF and participant characteristics (exercise-group, age, waist circumference, height, weight, BMI, training years and high intense activity) were analyzed by multiple linear regressions using backward elimination. Type II semi-partial correlation coefficients are reported for significant predictors. These represent the additional explanatory power of the given parameter after accounting for the other parameters in the model. Possible differences in the rate of change with age between athletes and controls were analyzed by a two-way (age and exercise-group) mixed model ANOVA with interactions.

Relations of SAF to diet and age were analyzed by simple regressions (both Pearson and Spearman). In addition, a similar multiple linear regression model as above was used to investigate potential relationships between SAF and dietary parameters (fruit, vegetables, fish, bread, cereals, coffee, wine, beer, liqueurs, total fluid, pure water, “Western” diet currently and “Western” diet prior to 18 years), the model also included age and exercise-group as explanatory variables.

The same analyses were performed with tendon thickness or Doppler area as dependent variable instead of SAF, with the addition of SAF to the list of explanatory variables in order to investigate possible effects of glycation on tendon structure. Statistical analyses were performed using Stata 15.1 (StataCorp LCC, College Station, TX, USA), and SAS 9.2 (SAS, Cary, NC, USA). A significance level of *p* < 0.05 was used. 

## 3. Results

### 3.1. Participant Characteristics

Basic participant characteristics are listed in Table 1. The controls were younger and heavier, with greater waist circumference and BMI than the athletes.

Mean dietary intakes for the two groups are listed in Table 2. The athletes consumed more fruit, vegetables and fish than the controls and reported a lower percentage of “Western” diet, both currently and in their youth. Consumption of grain products and liquids were similar in both groups.

### 3.2. SAF

In relation to diet, the simple regressions of SAF on each dietary parameter (Table 3) showed that SAF increased with age and with greater consumption of coffee, wine, and alcohol, while it decreased with greater water consumption. These factors were significant using both parametric Pearson and non-parametric Spearman correlation (Table 3). In multiple regression on the dietary parameters, none of the simple correlates except for age were significant independent predictors of SAF, but the fraction of “Western” diet during youth was identified as a significant predictor of increased current SAF levels in adulthood (*p* = 0.021) (Table 4).

**Figure 1:**
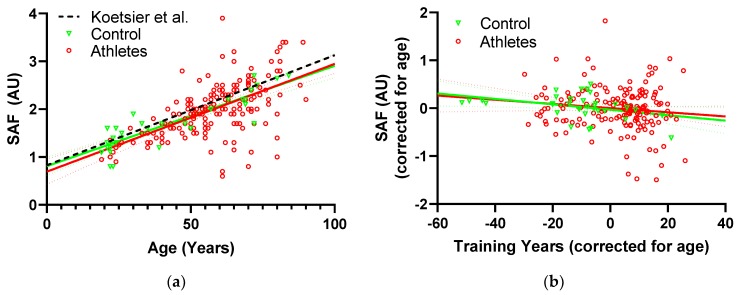
(**a**) SAF as a function of age in controls (green) and athletes (red) with associated linear regressions and 95% confidence intervals. The fit obtained by Koetsier et al. [33] on a general non-smoking population is included for comparison (black), (**b**) SAF as a function of training years after correcting for age. Values on the x- and y-axes represent residuals after regression to age (i.e., for each participant how much lower/higher was the value than expected from their age). Controls (green) and athletes (red) with associated linear regressions and 95% confidence intervals.

The relationship between SAF and age in both the athletes and controls is shown in Figure 1a. Multiple linear regression against participant characteristics (Table 4) demonstrated that SAF increased with age (*p* < 0.0001) and decreased with training years (*p* = 0.041). The relation of SAF to training years is illustrated in Figure 1b. In the two-way mixed model of SAF versus age and exercise-group, there was no significant interaction (*p* = 0.70), indicating that the accumulation rate did not differ between the athletes and controls.

### 3.3. Tendon Structure

**Figure 2:**
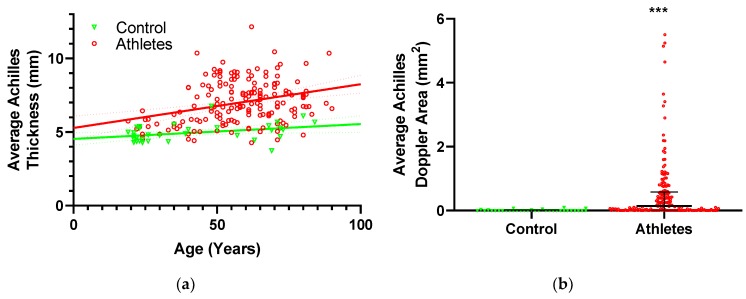
(**a**) Relationship between average Achilles tendon thickness and age in controls (green) and athletes (red) with associated linear regressions and 95% confidence intervals, (**b**) Achilles tendon vascularity quantified as the area containing detectable Doppler flow within the tendon. Data are displayed with median and interquartile range. Difference from control: *** *p* < 0.001.

Achilles tendon thickness as a function of age is displayed in Figure 2a. Multiple regression of Achilles tendon thickness on participant characteristics (Table 4) identified increased age (*p* < 0.0001) and height (*p* = 0.0075) as well as being in the athletic group (*p* < 0.0001), as significant predictors of greater thickness. In the two-way mixed model of thickness versus age and exercise-group, there was a trend to interaction, but it was not significant (*p* = 0.10), indicating that the rate of thickness increase with age did not differ significantly between the athletes and controls. The regression model on dietary parameters only had age and exercise group as significant predictors of thickness (same as in the analysis of participant characteristics above), indicating that diet did not affect tendon thickness.

Performing the same analyses on vascular Doppler flow (Table 4) showed that Doppler was greater in athletes than controls (*p* = 0.0023). Due to the highly skewed distribution of Doppler values (Figure 2b), a comparison of Doppler between athletes and controls was made using the non-parametric Mann–Whitney test, which also revealed significantly greater Doppler in the athletes (*p* < 0.0001). Similar to thickness, the regression model on dietary parameters only had exercise-group as a significant contributor, suggesting that diet did not affect tendon vascularity.

## 4. Discussion

To our knowledge, this is the first study that has investigated associations of long-term training, skin autofluorescence (SAF) and Achilles tendon structure in healthy adult men. Contrary to our expectations, there was no difference in SAF between the athletes and controls, however, more training years was independently associated with lower SAF. Diet also had an effect, with a more “Western” diet in youth being associated with increased SAF. Furthermore, our data demonstrated that greater Achilles tendon thickness was associated with aging and training. Together, our data indicate that long-term exercise is associated with lower glycation and greater Achilles tendon size, which may protect against injury.

### 4.1. SAF and Diet

The dietary pattern of the athletes differed significantly from that of the controls, with athletes consuming more fruit, vegetables, and fish, as well as reporting a less “Western” dietary pattern. Overall the athletes, therefore, consumed what could be categorized as a healthier diet. This relation between diet and exercise-group is critical to keep in mind when examining the effects of these parameters, and therefore, exercise-group was included in the multiple regression models for diet.

Several of the recorded dietary fluids displayed simple correlations to SAF. Coffee, wine, and alcohol consumption were positively related to SAF while intake of pure water was negatively related. A relationship between coffee and SAF has been consistently observed in several previous studies on both healthy and diabetic persons [29,34,35]. The magnitude of the effect is equivalent to approximately 2.6 years of aging per daily cup, which is comparable to the value reported by van Waateringe et al. [29]. Lists of AGE content in various food items have been reported, based either on assays using antibodies [36] or more recently by mass spectrometry [37,38]. Discrepancies exist between the two methods, but both suggest that the AGE content in coffee is tens to hundreds of times lower than most solid food products. The consistently reported association between SAF and coffee is, therefore, a bit surprising. While AGE levels in coffee may not be as high as most solid foods, it is at the high end of the reported beverages especially if exposed to prolonged heating [36]. The AGEs found in beverages are necessarily in a soluble form, and there is evidence to suggest that this increases bioavailability compared to AGEs from solid foods [39], which could contribute to the seemingly more significant influence of coffee on SAF compared to foods with higher AGE contents.

To our knowledge water consumption has not previously been assessed with respect to SAF, but it seems likely that reduced SAF with increasing water intake could result from the replacement of other AGE or sugar-containing beverages with water. Alcohol and wine have previously been associated with reduced SAF, but these findings seem less consistent than for coffee [40,41]. In the present study, a positive correlation was found, so opposite of what was previously reported, however, in multiple regression, none of the dietary fluids, including coffee, were significant contributors to SAF. This indicates that much of the relation was driven by age-related differences in diet. Overall, the present results, therefore, do not corroborate the previous studies in which coffee remained a significant contributor independently of age [29,34,35]. With age included in the model, a “Western” diet during youth was the only significant contributor to SAF, indicating that consuming a 100% “Western” diet would result in 0.3 units greater SAF than a 100% “Mediterranean” diet, equivalent to approximately 14 years of aging, however, it should be noted that the explanatory power is low, with an R^2^ of only 1.6% (Table 4). One of the key elements separating the two diet patterns is a greater consumption of meat in the “Western” diet, and meat consumption has previously been reported to positively correlate to SAF, which could, therefore, be a contributing factor [28].

The original idea behind including a measure of diet during youth was that the Achilles tendon is largely formed during this period with little to no further turnover during adult life [6], and consequently, youth conditions could impact the tendon in particular. However, the effect on SAF was a bit surprising since skin has greater turnover throughout life and we, therefore, did not expect a particularly strong effect of conditions during youth, if anything we had expected current diet pattern to be more influential. It should also be noted that the diet pattern during youth was self-reported, based on recollection dating back several decades for many of the participants.

### 4.2. SAF, Exercise and Aging

It is well established that SAF increases with chronological age, in agreement with the present work [2,33]. Less is known about the effects of training on SAF. In a smaller sample, we have previously demonstrated that life-long endurance running is associated with lower SAF [16]. Further, we have preliminary data to suggest that elderly life-long elite badminton players have lower SAF on their dominant playing arm than their non-dominant arm, but this was not true in younger elite badminton players. A rodent model has also reported lower plasma levels in protein-linked autofluorescence and carboxymethyl lysine with long-term exercise [42]. Studies in humans suggest that glycation also has negative effects on the ability to perform exercise, showing an inverse relationship between SAF and grip strength as well as leg extension power [43,44]. In the present study, we found contrary to our expectations, no significant difference in SAF between the athletes and controls, however, greater duration (training years) of exercise was independently associated with lower SAF. Looking at the coefficients, the reduction with one training year is approximately 18% of the yearly increase, suggesting that an active year contributed 18% less SAF than an inactive year. The strength of this association was fairly weak with an R^2^ of only 1.2%, but it is worth noting that it appears to be present in both athletes and controls and is therefore not driven by the difference in training years between the exercise groups (Figure 2b). Although exercise did not have the expected effect on skin AGEs, it is still possible that tissues experiencing direct mechanical stimulation, such as the Achilles tendon, may be affected due increased turnover [45]. Collectively, these data indicate that long-term exercise may be required to affect SAF levels in collagen-rich tissues such as skin. It is also worth noting that the controls in the present study have a bit lower SAF values than those previously reported in a general population (Figure 1a) [33], and our controls may have been more physically active than average (Table 1).

### 4.3. SAF and Biometrics

None of the biometric factors (waist circumference, height, weight, BMI) contributed significantly to SAF in the present study. This is somewhat contrary to previous studies that have reported positive correlations of SAF with waist circumference, weight and BMI [28,40,46]. These findings suggest a relation between SAF and adiposity, however, it appears likely that the effect is indirect, possibly mediated by pathways of metabolic disease. If that is the case, it could explain why these parameters were not significantly associated in the present study population, which consisted of fairly healthy persons. This is supported by a study reporting that waist circumference was only significantly related to SAF in centrally obese but not non-obese persons [40]. 

### 4.4. Tendon Structure, SAF and Aging

Glycation has been shown to induce collagen swelling at the molecular level [9,10], and previous studies have reported increased thickness of the tendon-like plantar fascia in diabetics showing a relation between thickness and diabetic complications [18,19,47]. However, it is not clear if these effects are mediated directly by glycation. Our initial hypothesis was therefore that greater SAF values would be associated with larger Achilles tendon thickness, however, SAF was not a significant predictor of tendon thickness in the present study.

In addition to swelling, glycation has been reported to reduce inter-fibrillar sliding capacity in animal tendon models [9,11,12]. Sliding between tendon layers has been shown to occur within the healthy Achilles tendon [48] and reduced intra-tendinous sliding capacity has been reported in both human and horse tendon with aging, which could be related to glycation [49,50]. Consequently, a relation between SAF and Doppler flow could be mediated through glycation related loss of sliding and subsequent pathology, but no such relation was observed in the present study. This finding is in agreement with previous work that did not find a relation between SAF and tendon abnormalities in type 1 and 2 diabetics [51]. 

### 4.5. Tendon Structure and Diet

Obesity and metabolic disease are risk factors for tendon injury [52,53,54], however fairly little is known about direct effects of diet on tendon. At the basic level, a number of micro-nutrients are known to influence important pathways in collagen formation, such as vitamin C being involved in proline hydroxylation and copper being required for covalent cross-linking by lysyl oxidase enzymes [55,56]. However, the present study made no attempt to determine micro-nutritional diet composition. At a macro-nutritional level, a study in humans reported that leucine-rich protein supplementation during an exercise intervention resulted in greater hypertrophy of both tendon and muscle [57], which is supported by a study on nutritional recovery in rats, which reported increased tendon collagen synthesis with a leucine-rich diet [58]. These results suggest that a protein-rich diet could result in tendon hypertrophy, perhaps especially during youth when the tendon is formed. However, the present study found no relation between tendon thickness and consumption of meat-rich “Western” diet, neither at present nor during youth.

Due to the limited turnover of tendon collagen, modification by glycation is of particular interest as it may accumulate to a large extent. In animals, greater dietary AGE intake has been reported to increase AGE content in tendons [14,59] and in addition, diet restriction has been found to reduce tendon AGE accumulation [60,61]. These findings indicate that AGEs in diet can affect the tendon. In animal studies, increased tendon mechanical stiffness or strength has also been reported in groups with higher tendon AGE content [14,60]. The possible effects of diet on tendon structure are not clear but, as mentioned previously, glycation may cause swelling and diabetes has been associated with increased tendon thickness. However, none of the animal models on dietary AGEs found any difference in tendon size [14,60]. This is in agreement with the present work in humans where none of the dietary parameters were associated with tendon thickness.

Although it is a different pathway than dietary AGEs, glycation has also been associated with reduced healing capacity in rats on a high glucose diet [62]. Since vascularization observable by Doppler is a key aspect of tendon pathology, dietary effects on healing could be associated with tendon Doppler, but no dietary association with Doppler was observed in the present study.

### 4.6. Tendon Structure and Exercise

Previous studies have reported increases in tendon size with exercise [63,64]. In untrained persons, increased tendon size has also been observed with aging [65], and old life-long endurance athletes appear to display an additive effect of exercise and aging, resulting in further increased tendon size than both age-matched sedentary controls and young athletes [63,66]. The present study corroborated those findings since both age and exercise group were significant independent predictors of thickness. A greater tendon size will reduce tendon stress, which may be a compensatory mechanism for lower material properties found with aging [65,67], thereby improving the tendon safety factor to reduce the risk of injury.

Higher Achilles tendon vascularity (Doppler) has also been reported with aging and years of badminton competition, indicating that greater tendon loading is associated with more vascularization in the tendon [32]. In the present study, despite athletes having more Doppler flow in their Achilles tendon, no associations could be demonstrated between Achilles tendon Doppler activity, aging, and years of exercise.

### 4.7. Tendon Structure and Biometrics

The Achilles tendon thickness was positively related to height, which is most likely just an expression of scaling to body size. Neither tendon thickness nor Doppler was related to any of the other biometric parameters. Theoretically, it could have been expected that greater weight or BMI would result in greater tendon load and consequently a larger tendon, in addition, there is some evidence to suggest that measures of adiposity (BMI, waist circumference) are connected to a higher prevalence of tendon pathology [52,53,54]. As mentioned previously, Doppler is associated with tendon pathology and an indirect link from adiposity to Doppler could, therefore, be possible [68]. The mechanisms responsible for the reported association of tendon pathology with measures of adiposity are unclear but could include direct effects of overloading due to increased weight, as well as systemic factors. If there were a direct effect of overloading, we suspect it would have been observed in the present study since, if anything, exercise activity would exacerbate such overload. However, numerous other factors must also be considered including the fact that biometric parameters were measured at the present time while tendon structural changes likely accumulate over many years (Figure 2a), and there may also be a certain “survivor” bias since persons experiencing tendon injuries may not remain physically active at the level required to be included in this study. The present findings, therefore, do not support a link between adiposity and tendon structure in long-term physically active persons, but additional work with a more specific focus on these issues would be required to fully elucidate the question.

### 4.8. Limitations

Being a cross-sectional study, the causal relations in the findings cannot be unambiguously determined. Due to the setting at a major sporting event, the extent of testing and the length of the questionnaire had to be limited, and consequently, the individual items such as the diet questionnaire were not comprehensive. The recruitment process made it impossible to blind investigators to the participants’ exercise group. While current smokers were excluded, several (*n* = 42) of the participants had been smoking previously in their lives, and there was an effect of prior smoking independent of age, with previous smokers having SAF values 0.22 AU greater than never-smokers (*p* = 0.004), equivalent to approximately 10 years of aging. The group of athletes recruited at EMACS had a wide range of nationalities whereas the controls were all Danish, differences related to nationality could, therefore, affect the analysis in relation to exercise-group. Skin pigmentation was not taken into account in this study, even though it may have an effect on the AGE Reader. The present model utilizes an algorithm to correct for skin pigmentation, but in a few participants we were unable to get a valid recording. While the controls were required to not have trained regularly in the past 10 years, many of them had been physically active for a number of years prior to that and as a consequence the control group may not be considered truly sedentary. Ultrasonography has been suggested to be less reliable to assess tendon structural properties compared to Magnetic Resonance Imaging, which for example makes it more difficult to measure tendon cross-sectional area, and therefore only tendon thickness was used. Age and exercise-related injury could affect the ultrasound structure, and while a detailed analysis of clinical and injury-related outcomes are beyond the scope of the present study, we did a simple analysis to support the validity of the present findings. Including tendon impairment at the time of examination, assessed by the Victorian Institute of Sports Assessment (VISA) questionnaire, in the model showed a significant relation to tendon structure, with greater impairment (lower score) resulting in greater thickness and Doppler. However, the other independent predictors reported in the present study were not substantially affected by the inclusion of VISA and remained significant. In addition, a simple assessment of previous overuse injuries to the Achilles found no effect on tendon structure, nor was there any relation between current (VISA) and previous tendon injuries on SAF.

## 5. Conclusions

In relation to the original hypotheses, our findings show that: (A) There was no significant difference in SAF between athletes and controls, but an association with training years, indicating that exercise may have a modest influence on glycation. (B) The only dietary parameter that was significantly associated with SAF was the consumption of a “Western” type diet during youth. (C) No association between SAF and tendon structure was observed. D) Our data demonstrate that Achilles tendon thickness and vascularity were greater in healthy athletes compared to controls, and that thickness increases with age in athletes. Together, these results indicate that athletes that have performed exercise for more than 10 years do not have lower glycation rates than controls, but that routine athletes have greater Achilles tendon size than sedentary controls, which may protect against injury.

## Figures and Tables

**Table 1:** Baseline characteristics.

	Controls	Athletes	Combined
Age (year)	42 ± 22	57 ± 15 ***	54 ± 18
Waist (cm)	89.7 ± 11.8	82.7 ± 6.2 **	83.9 ± 7.8
Height (cm)	180.3 ± 5.5	178.2 ± 6.7	178.6 ± 6.6
Weight (kg)	81.0 ± 12.3	73.8 ± 8.5 **	74.9 ± 9.6
BMI (kg/m2)	25.0 ± 3.8	23.2 ± 2.2 *	23.5 ± 2.6
Training years (years)	13 ± 18	36 ± 15 ***	32 ± 18
High intense activity (hours/week) ^1^	0.17 ± 0.38	4.57 ± 4.04 ***	3.86 ± 4.04

^1^ Self-reported current activity at >70% of max heart rate. Data are given as mean ± SD. Difference from controls: * *p* < 0.05, ** *p* < 0.01, *** *p* < 0.001.

**Table 2:** Mean weekly dietary intake.

	Controls	Athletes
Fruit (pieces)	7.9 ± 7.1	15.3 ± 9.8 ***
Vegetables (100g portions)	9.7 ± 9.0	13.5 ± 8.7 *
Fish (100g portions)	1.2 ± 1.6	2.1 ± 3.4 *
Rye or wholegrain bread (slices)	16 ± 11	16 ± 12
Oat or wholegrain cereals (1 dL servings)	4.1 ± 5.2	5.7 ± 5.9
Coffee (cups)	16 ± 14	16 ± 12
Wine (glasses)	5.0 ± 9.5	4.2 ± 8.0
Beer (bottles)	4.2 ± 7.8	2.6 ± 5.8
Liquors and spirits (drinks)	1.6 ± 4.2	0.4 ± 2.2
Total fluid (liters)	17.3 ± 5.5	18.0 ± 6.9
Pure water (liters)	9.1 ± 5.6	9.8 ± 5.8
“Western” diet currently (%)	46 ± 25	28 ± 21 ***
“Western” diet prior to 18 years (%)	56 ± 21	44 ± 25 **

Data are given as mean ± SD. Difference from controls: * *p* < 0.05, ** *p* < 0.01, *** *p* < 0.001.

**Table 3:** Coefficients of simple linear regression between SAF and dietary parameters in the complete study population.

	Pearson					Spearman	
	Coefficient	R^2^	*p*-Value	95%-CI		rho	*p*-Value
Age (year)	0.022	0.441	<0.001 ***	0.018	0.025	0.681	<0.001 ***
Fruit (pieces)	0.001	<0.001	0.841	−0.007	0.009	0.055	0.427
Vegetables (100g portions)	−0.006	0.008	0.193	−0.015	0.003	−0.056	0.422
Fish (100g portions)	0.018	0.011	0.138	−0.006	0.043	0.112	0.105
Rye or wholegrain bread (slices)	0.0004	<0.001	0.896	−0.006	0.007	0.025	0.722
Oat or wholegrain cereals (1 dL servings)	−0.009	0.009	0.186	−0.023	0.004	−0.076	0.273
Coffee (cups)	0.008	0.033	0.008 **	0.002	0.014	0.229	<0.001 ***
Wine (glasses)	0.013	0.036	0.006 **	0.004	0.023	0.215	0.002 **
Beer (bottles)	−0.007	0.005	0.310	−0.020	0.006	0.011	0.881
Liquors and spirits (drinks)	0.035	0.026	0.021 *	0.005	0.064	0.146	0.036 *
Total fluid (liters)	−0.009	0.012	0.119	−0.021	0.002	−0.132	0.063
Pure water (liters)	−0.015	0.024	0.027 *	−0.029	−0.002	−0.182	0.009 **
“Western” diet currently (%)	−0.001	0.002	0.512	−0.005	0.002	−0.083	0.233
“Western” diet prior to 18 years (%)	−0.001	0.001	0.609	−0.004	0.002	−0.039	0.573

Significance levels: * *p* < 0.05, ** *p* < 0.01, *** *p* < 0.001. 95%-CI = 95% confidence interval.

**Table 4:** Result of backward elimination multiple regression.

		Coefficient	SE	*p*-Value	Semi-Partial R^2^ (%)
**SAF (AU).** Model parameters: Age, exercise-group, waist circumference, height, weight, BMI, training years and high intense activity.					
Resulting model: *n* = 201, *p* < 0.0001, R^2^ = 45.9%.					
	Intercept	0.728	0.099	<0.0001	
	Age (years)	0.0248	0.0022	<0.0001	35.1
	Training years (years)	−0.0044	0.0021	0.041	1.2
**SAF (AU).** Model parameters: Age, exercise-group, fruit, vegetables, fish, bread, cereals, coffee, wine, beer, liqueurs, total fluid, pure water, “Western” diet currently and “Western” diet prior to 18 years.					
Resulting model: *n* = 195, *p* < 0.0001, R^2^ = 42.7%.					
	Intercept	0.58	0.13	<0.0001	
	Age (years)	0.0219	0.0018	<0.0001	42.7
	“Western” diet prior to 18 years (%)	0.0031	0.0013	0.021	1.6
**Achilles tendon thickness (mm).** Model parameters: Age, exercise-group, waist circumference, height, weight, BMI, training years, high intense activity and SAF.					
Resulting model: *n* = 197, *p* < 0.0001, R^2^ = 33.8%.					
	Intercept	−3.6	2.8	0.21	
	Age (years)	0.0288	0.0059	<0.0001	8.2
	Exercise-group (athlete)	1.64	0.25	<0.0001	14.8
	Height (cm)	0.041	0.015	0.0075	2.5
**Achilles tendon Doppler (mm^2^)** Model parameters: Age, exercise-group, waist circumference, height, weight, BMI, training years, high intense activity and SAF					
Resulting model: *n* = 197, *p* = 0.0023, R^2^ = 4.65%.					
	Intercept	0.01	0.16	0.94	
	Exercise-group (athlete)	0.53	0.17	0.0023	4.65

SE = standard error.

## Supplementary Materials

The following are available online at https://www.mdpi.com/2072-6643/11/6/1409/s1.
