# Supplementary material for: Effects of Long-Term Physical Activity and Diet on Skin Glycation and Achilles Tendon Structure
Source: Nutrients. 2019 Jun 22;11(6):1409. doi: 10.3390/nu11061409 (PMC6627972; doi:10.3390/nu11061409)
Supplement: Supplementary file 1 [file nutrients-11-01409-s001.pdf]

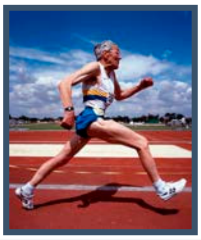

SECTION FOR SPORT SCIENCE
DEPARTMENT FOR PUBLIC HEALTH
AARHUS UNIVERSITY

Questionnaire

FP-ID:______________

Name:____ Date:__________

E-mail:____

Mobile:____

Nationality:____

Age: ________

Height (cm):___________

Weight (kg):____________

Waist (cm):_____________

EMACs 2017

Which competitions have you or are you going to participate in:

Date:____

Date:____

Date:____

Date:____

Christian Couppé, Senior Researcher, PhD
Center for Injury Prevention and Protection of Athlete Health
Bispebjerg Hospital, Bispebjerg Bakke 23
2400 Copenhagen NW

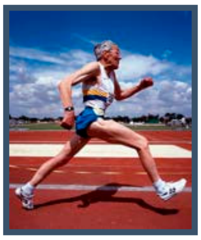

Sports activities (total)

Hours per week with low/moderate intensity physical activities (walking, light gymnastic etc

< 70% of max heartrate)____(write number of hours)

Hours per week with high intensity physical activities (such as running, sport cycling, aerobic

etc. > 70% af max heartrate______________________________________(write number of hours)

For how many years during your life have you been regularly physically active at least two

times per week?___________________________________

Christian Couppé, Senior Researcher, PhD
Center for Injury Prevention and Protection of Athlete Health
Bispebjerg Hospital, Bispebjerg Bakke 23
2400 Copenhagen NW

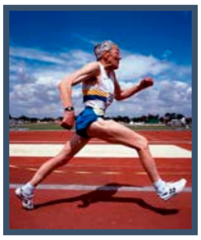

Diet:

We will now ask you questions related to your diet. Your answers should reflect your diet in the last 3 months up to today.

1. How often do you eat **fruit?**

____Less than 1 piece / week

____ 1-2 pieces / week

____ 3-4 pieces / week

____ 5-6 Pieces / week

____ 1 piece / day

____ 2-3 pieces / day

____ 4-5 pieces / day

____ more than 6 piece / day)

2. How often do you eat **vegetables** (1 portion = about 100g) (potatoes are not included)

____ Less than 1 portion / week

____ 1-2 portions / week

____ 3-4 portions / week

____ 5-6 portions / week

____ 1 portion / day

____ 2-3 portions / day

____ 4-5 portions / day

____ more than 6 portions / day)

3. How many times a week do you eat **fish?** (1 portion 100 g)

____ Less than 1 portion / week

____ 1-2 portions / week

____ 3-4 portions / week

____ 5-6 portions / week

____ 1 portion / day

____ 2-3 portions / day

____ 4-5 portions / day

____ more than 6 portions / day)

4. How many sliced **rye bread / whole grain bread** do you eat?

____ Less than 1 slice / week

____ 1-2 slices/ week

____ 3-4 slices / week

____ 5-6 slices / week

____ 1 slice / day

____ 2-3 slices / day

____ 4-5 slices / day

____ more than 6 slices / day)

Christian Couppé, Senior Researcher, PhD
Center for Injury Prevention and Protection of Athlete Health
Bispebjerg Hospital, Bispebjerg Bakke 23
2400 Copenhagen NW

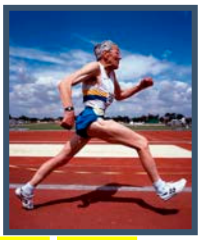

5. How often do you eat oat/whole grain cereals (1 serving = 1 dl)?

____ Less than 1 portion / week
____ 1-2 portions / week
____ 3-4 portions / week
____ 5-6 portions / week
____ 1 portion / day
____ 2-3 portions / day
____ 4-5 portions / day
____ + 6 portions / day)

6. How many cup of coffee?

____Less than 1 cup / week
____ 1-2 cups / week
____ 3-4 cups / week
____ 5-6 cups / week
____ 1 cups/ day
____ 2-3 cups / day
____ 4-5 cups / day
____ + 6 cups / day)

7. How many glasses of wine?

____Less than 1 glas / week
____ 1-2 glasses / week
____ 3-4 glasses / week
____ 5-6 glasses / week
____ 1 glasses/ day
____ 2-3 glasses / day
____ +4 glasses / day

Christian Couppé, Senior Researcher, PhD
Center for Injury Prevention and Protection of Athlete Health
Bispebjerg Hospital, Bispebjerg Bakke 23
2400 Copenhagen NW

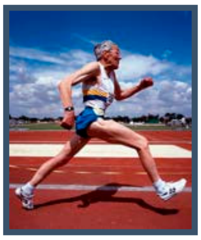

8. How many bottles of beer?

____Less than 1 bottle / week

____ 1-2 bottles / week

____ 3-4 bottles / week

____ 5-6 bottles / week

____ 1 bottle / day

____ 2-3 bottles / day

____ +4 bottles / day

9. How many drinks of liquors and spirits?

____Less than 1 drink / week

____ 1-2 drinks / week

____ 3-4 drinks / week

____ 5-6 drinks / week

____ 1 drink / day

____ 2-3 drinks / day

____ +4 drinks / day

Total liters fluid per day?__________and how many liters are pure water? ______________________

____

Christian Couppé, Senior Researcher, PhD
Center for Injury Prevention and Protection of Athlete Health
Bispebjerg Hospital, Bispebjerg Bakke 23
2400 Copenhagen NW

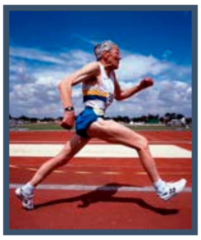

Description of your current diet pattern

Below are two description of two diet patterns. How well do each of them **describe your current diet habits** (the average over the last 3 months).

**Diet pattern A**	**Diet pattern B**
• Dense in energy<br>• Rich in fat (butter)<br>• Rich in red meat<br>• Typical refined cereals rich in sugar<br>• Food products rich in sugar and fat and salt<br>• Often intake of soft drinks<br>• Candy bars, desserts, sweet bakery goods, savoury snakcks<br>• Deep-fried potatoes, savoury snacks, etc.	• Rich in plant foods<br>• Rich in fresh fruit<br>• Plant oil primary fat source (e.g. olive oil and rapeseed oil)<br>• Pulses<br>• Cheese, yoghurt<br>• Fish at least two times a week<br>• Low to moderate consumption of wine<br>• Only small amounts of red meat.<br>• Only whole grain cereals/bread

0% = does not describe my diet habits at all. 100% = fits 100% with my diet habits.

Diet pattern A correspond to:________________% of my diet, and

Diet pattern B correspond to:________________% of my diet (In total: A + B = 100%)

Description of your previous dietary pattern (0-17 years old)

How well does each of the descriptions above **describe your dietary habits** **until you became 17 yrs old**

Diet pattern A correspond to:________________% of diet before the age of 17 years, and

Diet pattern B correspond to:________________% of diet before the age of 17 years (In total: A + B = 100%)

Christian Couppé, Senior Researcher, PhD
Center for Injury Prevention and Protection of Athlete Health
Bispebjerg Hospital, Bispebjerg Bakke 23
2400 Copenhagen NW

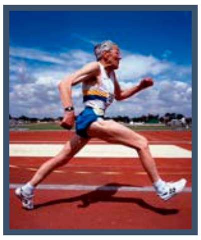

Smoking: No, never_______________________

No, but were smoking 0-20 years ago________________ Duration_______________yrs

No, but were smoking more than 20 years ago _______ Duration______________ yrs

Yes, 0-7 cigarettes per week (1 cigarette per day)

Yes, 8-21 cigarettes per week (2-3 cigarettes per day)

Yes, more than 22 cigarettes per week (more than 3 cigarettes per day)

Christian Couppé, Senior Researcher, PhD
Center for Injury Prevention and Protection of Athlete Health
Bispebjerg Hospital, Bispebjerg Bakke 23
2400 Copenhagen NW
